# Integrative Dissection of Novel Lactate Metabolism-Related Signature in the Tumor Immune Microenvironment and Prognostic Prediction in Breast Cancer

**DOI:** 10.3389/fonc.2022.874731

**Published:** 2022-04-27

**Authors:** Lu Yang, Peixin Tan, Hengwen Sun, Zijun Zeng, Yi Pan

**Affiliations:** Department of Radiation Oncology, Guangdong Provincial People’s Hospital, Guangdong Academy of Medical Sciences, Guangzhou, China

**Keywords:** lactate metabolism, breast cancer, prognostic signature, tumor immune microenvironment, immunotherapy

## Abstract

The outcomes of some breast cancer patients remain poor due to being susceptible to recurrence, metastasis and drug resistance, and lactate metabolism has been described as a hallmark of cancer and a contributor to cancer progression and immune escape. Hence, it is worthy of seeking potentially novel biomarkers from lactate metabolism relevant perspectives for this particular cohort of patients. In this context, 205 available lactate metabolism-related genes (LMGs) were obtained by a search of multiple genesets, and the landscape of somatic mutation, copy number variation, and mRNA expression levels was investigated among these genes. Crucially, 9 overall survival-related LMGs were identified through univariate Cox regression analysis in The Cancer Genome Atlas (TCGA) and Molecular Taxonomy of Breast Cancer International Consortium (METABRIC) databases. Subsequently, a prognostic signature, defined as Lactate Metabolism Index (LMI), was established with 5 OS-related LMGs using Least Absolute Shrinkage and Selection Operator (LASSO) Cox hazard regression analysis in TCGA training set, and then validated in two external cohorts (METABRIC and GSE96058). From the comprehensive results, breast cancer patients with high LMI had considerably poorer survival probability across all cohorts, and the degree of clinical features tended to be more severe as the LMI value increased. Furthermore, a prognostic nomogram incorporating LMI, age, and AJCC stage was constructed and demonstrated great prediction performance for OS of breast cancer patients, which was evaluated by the calibration plot and the decision curve analysis. Moreover, the potential effect of different LMI values on levels of immune checkpoints, tumor-infiltrating immune cells, and cytokines were explored ultimately, and patients with higher LMI values might gain an immunosuppressive tumor microenvironment that contributed to immune escape of breast cancer and inferior prognosis. Collectively, all findings in the study indicated the potential prognostic value of LMI in breast cancer, providing further implications for the role of lactate metabolism in breast cancer prognosis, tumor immune microenvironment, and immunotherapy.

## Introduction

Owing to high morbidity and corresponding mortality in women, breast cancer (BC) has consistently received extensive attention worldwide (1). Also, with the development of comprehensive therapy strategies, outcomes for patients with breast cancer have extremely improved. However, the outcomes of some patients are still poor due to being susceptible to recurrence, metastasis, and drug resistance (2). Thus, there is still a need to find novel and effective biomarkers to identify this subgroup of patients with breast cancer.

In recent years, it is becoming increasingly evident that lactate metabolism plays a critical role in tumor progression and has been a hallmark of cancer (3). Previous studies have shown that lactate has prognostic and predictive utility in several cancer types. For example, a higher lactate level was correlated with worse outcomes in cervical cancer (4). In head and neck squamous cell carcinoma, lactate was found to be inversely correlated with overall and disease-free patient survival and positively correlated with radioresistance (5, 6). For breast cancer, it has been reported that elevated intratumoral lactate levels were an adverse prognostic factor in breast cancer (7). Furthermore, raised intratumoural lactate levels were related to HER2 addiction status and trastuzumab susceptibility in HER2-positive breast cancer (8). Despite this, a comprehensive review of the influence of lactate on breast cancer was still lacking.

It is commonly accepted that the tumor microenvironment (TME), which included not only the surrounding stromal and immune cells but also the changes of metabolites and signaling molecules, has emerged as a major regulator to drive cancer development and progression (9). Lactate, produced by cancer cells, was secreted into the extracellular space and then functioned as a contributor to facilitating tumor immune escape (10). Specifically, lactate accumulation could directly inhibit the cytotoxic functions of T cells and innate lymphocytes such as natural killer (NK) and natural killer T (NKT) cells (11–13), induce a tolerogenic DC phenotype promoting regulatory T-cell (Treg) polarization (14), and cause accumulation and polarization of myeloidderived suppressor cells (MDSCs) and M2-tumor-associated macrophages (TAMs) (10, 15). Because of this immunosuppressive role, lactate played a negative role in the efficacies of immunotherapy. High lactate dehydrogenase (LDH) level was identified as an independent biomarker for predicting therapeutic response to immune checkpoint inhibitors (ICIs), including anti-PD-1 and anti-CTLA-4 therapy, in patients with melanoma, non-small cell lung cancer, and esophageal squamous cell carcinoma (16–18). However, a 2020 meta-analysis of ICIs in metastatic breast cancer did not find a utility for LDH in predicting response to these treatments (19). To further explore the immune-related role of lactate metabolism in breast cancer, a landscape assessment of the relationship between lactate metabolism and TME and ICIs therapy remains necessary.

In the current study, we aimed to screen out prognostic lactate metabolism-related genes (LMGs) and identify a prognostic signature based on LMGs for predicting the outcomes of patients with BC. In addition, the utility of the signature applied in the clinic was completely evaluated. Subsequently, the potential correlation between the signature and the landscape of TME was systematically dissected. The comprehensive analysis might provide more detailed insights into the cancer research about lactate metabolism and immunotherapy.

## Materials and Methods

### Data Collection and Acquisition of Lactate Metabolism-Related Genes

The public transcriptome expression matrices and detailed clinical data of BC were collected from The Cancer Genome Atlas (TCGA) database^1^ (113 normal breast samples and 1,109 BC samples), the Molecular Taxonomy of Breast Cancer International Consortium (METABRIC) database^2^ (1,904 BC samples), and GSE96058 in the Gene Expression Omnibus (GEO) database^3^ (3,409 BC samples), respectively. Besides, 1,082 BC patients of TCGA were selected as the training set, while 1,903 BC samples of METABRIC and 3,409 BC patients were chosen as external validation sets after excluding patients without overall survival information. In total, 284 lactate metabolism-related genes were acquired from the Molecular Signature Database v7.5.1 (MSigDB)^4^. Furthermore, 205 overlapping LMGs were screened out for further analyses after intersecting the above 284 LMGs with the total genes in TCGA-BRCA, METABRIC, and GSE96058 datasets (**Supplementary Figure S1**).

### Investigation of Somatic Mutation, Copy Number Variation Frequency and Differentially Expressed Genes Among LMGs

We obtained the information on the somatic mutation and copy number variation (CNV) of BC from TCGA-BRCA in the UCSC Xena database^5^. The somatic mutation frequency of 205 LMGs was analyzed by the R package “maftools” (20) and visualized in an oncoplot waterfall plot. Additionally, the CNV frequency of LMGs was calculated and visualized in a bi-directional column chart. The differentially expressed genes (DEGs) of LMGs were identified after comparing the normal breast samples and BC samples in TCGA-BRCA dataset with the threshold set to |log2FC| >1 and false-discovery rate (FDR) <0.05 using the “edgR” R package (21). A heatmap and a volcano plot of these significant DEGs were shown subsequently.

### Identification of Overall Survival-Associated LMGs

To elucidate the underlying prognostic significance of 205 LMGs in BC, overall survival (OS)-associated LMGs were identified through univariate Cox hazard regression analysis with *p* < 0.05 in the TCGA-BRCA (*n* = 1,082) and METABRIC (*n* = 1,903) cohorts, respectively (**Supplementary Tables S1****,** **S2**). Moreover, the overlapping OS-associated LMGs were extracted to further research. Meanwhile, the expression levels and the location on chromosomes of those eligible LMGs were illustrated by the “RCircos” R package (22), and the correlation characteristics among these LMGs were demonstrated in a correlation matrix plot.

### Construction and Validation of Lactate Metabolism-Correlated Prognostic Signature

Least Absolute Shrinkage and Selection Operator (LASSO) Cox regression analysis was performed in the training cohort to construct the statistical prognostic signature utilizing the candidate OS-related LMGs. Next, 5 optimal LMGs were dug out to establish the prognostic model for BC patients, while the expression levels and prognostic significance of each signature-contained LMG were depicted, respectively. In according with the predictive prognostic signature, Lactate Metabolism Index (LMI) could be calculated for each BC patient by employing the following formula:


LMI=∑Expression of Each LMG∗Corresponding Regression Coefficient

To make data and plots more intuitionistic, a linear transformation was carried out to adjust the LMI in each dataset using the following formula:


$$adj.LMI=\frac{LMI-min\left(LMI\right)}{\max\left(LMI\right)-\min\left(LMI\right)}$$

Afterwards the BC patients in each cohort could be separated into the high- and low-LMI groups by the cutoff of the median LMI value. Principal component analysis (PCA) was carried out to evaluate the classification accuracy of the signature. To discover the feasibility of the signature, a K-M analysis of OS was executed between the high- and low-LMI groups in three datasets separately.

### Integrated Dissection of LMI and Clinical Parameters in Patients With BC

To decipher the availability of LMI applied in actual clinical issues further, boxplots with the Kruskal test were exhibited to compare the distribution of adjusted LMI value in various degrees of diverse clinicopathologic parameters available in the three datasets. Besides, heatmaps were shown to unveil the relevance between each signature-included LMGs’ expression level and several clinical indicators, comprising of LMI, T stage, N stage, AJCC stage, PAM50 subtypes, and survival status in the training set in addition to LMI, tumor size, positive nodes, PAM50 subtypes, AJCC stage, and survival status in two validation datasets.

### Establishment and Evaluation of Lactate Metabolism-Correlated Clinical Nomogram

Subsequently, it was delineated whether the LMI was an independent prognostic predictor in BC by univariate and multivariate Cox regression analyses. Based on the results above, a lactate metabolism-correlated clinical nomogram that integrated LMI, age, and AJCC stage in TCGA-BRCA was established through the “rms” and “regplot” R packages (23). To assess the satisfactory predictive discrimination of nomogram, the calibration curve (24) and the decision curve analysis (DCA) plot were portrayed for BC patients.

### Clarification of Different Biological Functions Within Two LMI Groups

“GSVA” R package (25) was employed to clarify the different biological functions and signaling pathways between high- and low-LMI groups in the training set. “c2.cp.kegg.v7.5.1.symbols.gmt” [KEGG] was retrieved from MSigDB as the reference molecular signature database and the pathways with adjusted *p*-values <0.05 were considered significant. Ultimately, the most significant pathways were displayed in a heatmap.

### Potential Implications for Immunotherapy and Tumor-Immune Microenvironment Landscape Estimation Based on LMI

To verify the potential implications for immunotherapy based on LMI, the expression levels of several immunologic checkpoints, comprising PD-1, PD-L1, CTLA4, CD96, VSIR, and TIGIT, were compared between the high- and low-LMI groups using the Wilcox test.

To dissect the TME landscape between the two LMI subgroups, the ESTIMATE algorithm (26) was implemented to calculate the estimate scores, immune scores, and stromal scores for further predicting tumor purity and analyzing the TME. Moreover, the CIBERSORT deconvolution algorithm (27) was utilized to estimate the abundance of 22 tumor immune-infiltrating cell types in the training set.

Likewise, to investigate the correlation between LMI signature and cytokines in TME, several essential cytokines were picked out to make a comparison between high- and low LMI subgroups in expression levels, including IL-1B, IL-2, IL-6, IL-10, IL-18, TNF, IFNG, GZMA, and GZMB.

### Cell Lines and Cell Culture

Human breast cancer cell lines were purchased from the American Type Culture Collection. All cell lines were cultured following standard guidelines. All cell lines were maintained without antibiotics in an atmosphere of 5% CO_2_ and 99% relative humidity at 37°C. Cell lines were passaged for fewer than 6 months and were authenticated by short tandem repeat analysis. No mycoplasma infection was found for all cell lines.

### RNA Isolation and Quantitative Real-Time PCR Analysis

Total RNA of cells was obtained with RNA-Quick Purification Kit (ES-RN001, Shanghai Yishan Biotechnology Co., Shanghai, China). The quantitative real-time PCR (qRT-PCR) plate was employed from NEST (402301, Wuxi NEST Biotechnology Co., Jiangsu, China). The primer sequences are shown in **Supplementary Table S3**. RNA levels were determined by qRT-PCR in triplicate on a Bio-Rad CFX96 using the SYBR Green method (RR420A, Takara, Mountain View, CA, USA). The RNA levels were normalized against β-actin RNA using the comparative Ct method.

### Statistical Analysis

All statistical analyses were completed *via* R software (Version 4.0.2, http://www.R-project.org). The discrepancy in the expression level of signature-encompassed LMGs in normal breast and BC samples, ESTIMATE algorithm-calculated scores, checkpoints, and cytokines in the low- and high-LMI groups were detected by the Wilcox test. The comparison of each Kaplan–Meier (KM) curve that occurred in this study was accomplished by the log-rank test. Also, Kruskal tests were performed to discover the differences in adjusted LMI values in various clinical parameters. Univariate and multivariate Cox regression analyses were employed to screen out the OS-related LMGs and the independent prognostic indicators of OS for BC. The correlation matrix plot was portrayed under Spearman’s correlation test. Statistical significance was confirmed as *p*-value <0.05, and all *p*-values were bilateral.

## Results

### Identification of Prognostic Lactate Metabolism-Related Genes in Breast Cancer

**Figure 1:**
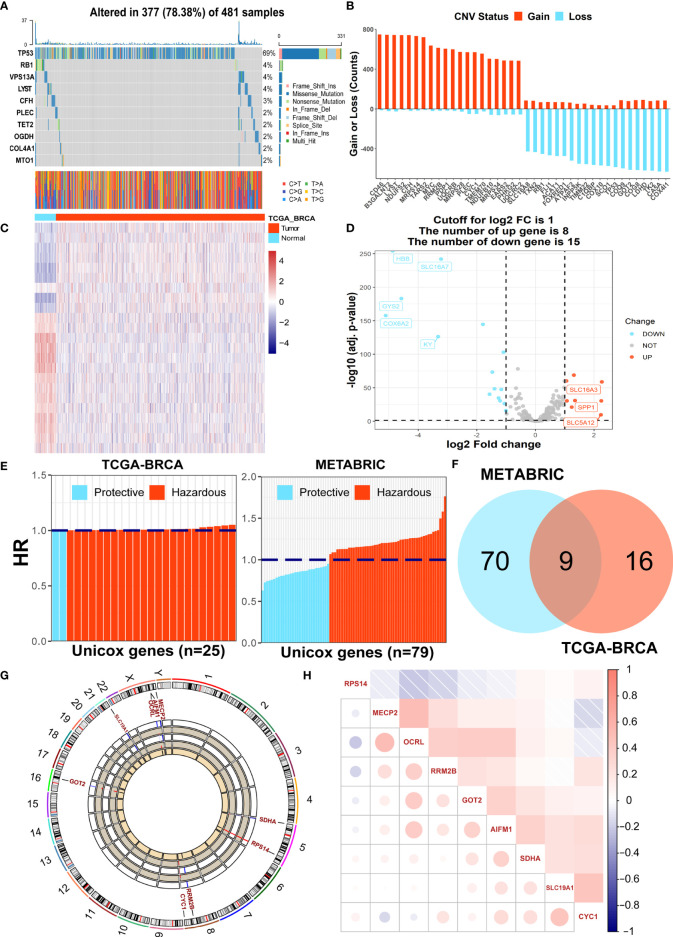
Identification of prognostic LMGs in BC patients. **(A)** The somatic mutation frequency of LMGs in the TCGA-BRCA cohort. **(B)** The prevalent CNV frequency of LMGs in the TCGA-BRCA cohort. **(C)** Heatmap of differentially expressed genes among LMGs. **(D)** Volcano plot exhibiting DEGs in LMGs. **(E)** OS-related LMGs in TCGA-BRCA and METABRIC datasets, respectively. **(F)** The Veen diagram to detect 9 common prognostic LMGs. **(G)** The Circos plot illustrating the locations on chromosomes and expression levels of the 9 candidate LMGs. **(H)** The correlation matrix plot containing 9 prognostic LMGs.

Initially, we assessed the global alterations of 205 LMGs in somatic mutation and copy number variation (CNV). As shown in **Figure 1A**, the top 10 genes with the highest somatic mutation rates were included in the heatmap, with the highest mutation frequency distributed in TP53. For frequency of CNV, the result showed that there were common CNV mutations among LMGs, and the top 20 genes in amplified and deleted CNV status separately were displayed in **Figure 1B**. Additionally, to investigate the differential expression of these LMGs, we compared their mRNA expression levels between 1082 tumor samples and 113 normal breast samples with the threshold of |log2FC| > 1 and FDR <0.05, and the results were demonstrated through a heatmap (**Figure 1C**) and a volcano plot (**Figure 1D**). Furthermore, to identify prognostic LMGs in breast cancer for the following research, the univariate Cox regression analyses were conducted to screen out OS-related genes in both TCGA and METABRIC datasets (**Figure 1E**). In total, 25 and 79 significant OS-related genes were obtained respectively and 9 overlapping genes (RPS14, MECP2, OCRL, RRM2B, GOT2, AIFM1, SDHA, SLC19A1, and CYC1) were included for further analysis after taking intersecting of the results above (**Figure 1F**). Moreover, the location of chromosomes and expression level of the 9 genes were illustrated by a Circos plot (**Figure 1G**). Finally, a correlation network plot was used to unravel the correlation features among 9 eligible FMGs (**Figure 1H**).

### Construction and Validation of Lactate Metabolism-Relevant Prognostic Signature for Patients With Breast Cancer

**Figure 2:**
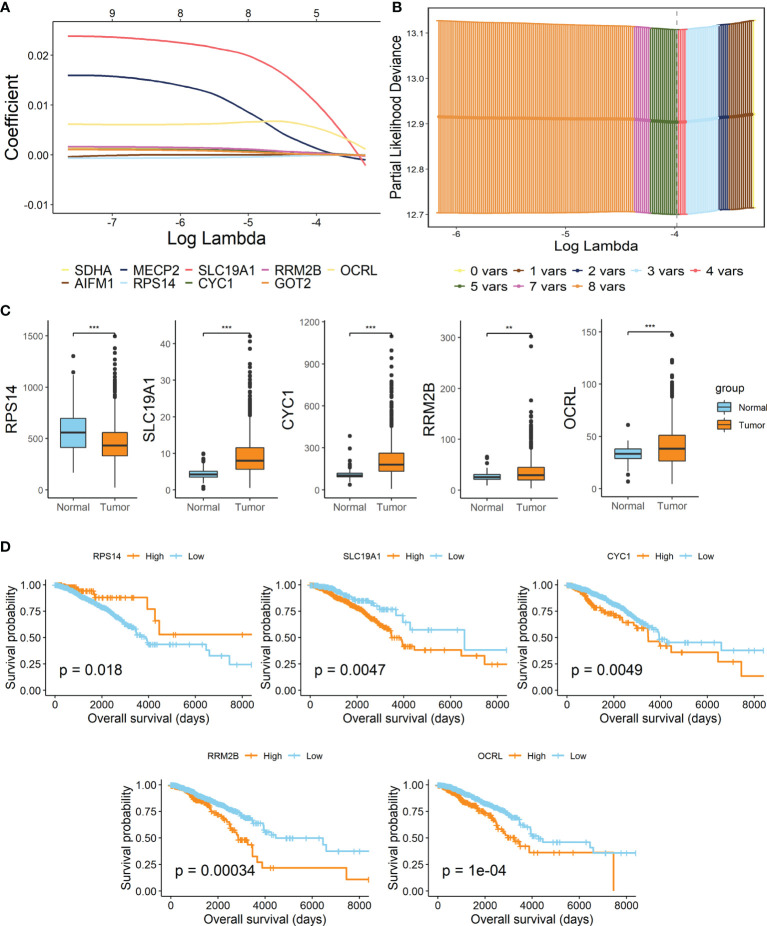
Establishment of lactate metabolism-related signature in BC. **(A)** LASSO Cox regression analysis. **(B)** Partial likelihood deviance for the LASSO regression to screen out 5 optimal prognostic LMGs. **(C)** Boxplots showing the mRNA expression levels of 5 signature-contained LMGs in the training cohort. **(D)** KM survival curves of OS based on expression levels of 5 LMGs in the training cohort. **p < 0.01; ***p < 0.001.

By performing the LASSO Cox regression analysis with 9 candidate genes in breast cancer patients of TCGA-BRCA training dataset, 5 pivotal genes were unearthed to establish the prognostic signature, Lactate Metabolism Index, namely LMI (**Figures 2A, B**), including RPS14, SLC19A1, CYC1, RRM2B, OCRL. Besides, the investigation of expression levels and survival capability of every signature-contained gene was further conducted by boxplots (**Figure 2C**) of mRNA expression levels and KM survival curves of OS (**Figure 2D**). From the results, we found that mRNA expressions of SLC19A1, CYC1, RRM2B, and OCRL were clearly elevated in breast cancer, while RPS14 expression was considerably decreased. Additionally, the RNA expression levels of SLC19A1 and RPS14 were validated in human breast cancer cell lines (**Supplementary Figure S2**), the result of which demonstrated that SLC19A1 was significantly promoted in breast cancer cell lines including MDA-MB-231, T47D, and SK-BR-3 while RPS14 declined significantly in BC cell lines comparing with breast epithelial cell line MCF10A. And for the survival analyses of OS, high expressions of SLC19A1, CYC1, RRM2B, and OCRL and downregulation of RPS14 were significantly related to more unfavorable OS in breast cancer, which further confirmed the validity of selected genes. Eventually, the prognostic signature was established as follows: LMI = Expression of SLC19A1 * 0.010382 − Expression of RPS14 * 0.000045 + Expression of CYC1 * 0.000408 + Expression of RRM2B * 0.000005 + Expression of OCRL * 0.006258.

### Validation of Signature Based on 5 LMGs

**Figure 3:**
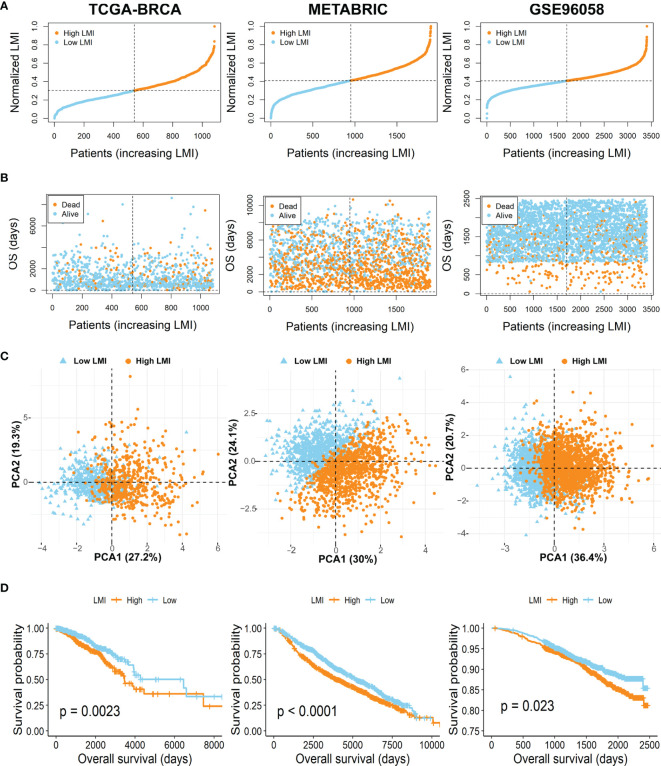
Evaluation and validation of the feasibility of LMI in training set and validation sets. **(A)** Distribution of the patients’ adjusted LMI score. **(B)** BC patients’ OS time along with their LMI score. **(C)** PCA analyses of high- and low-LMI groups. **(D)** KM curves for the OS of BC patients between high- and low-LMI groups.

To further verify the prognostic value of LMI in breast cancer, patients of the TCGA-BRCA training set and two validation sets (METABRIC and GSE96058) were then independently segregated into high-LMI and low-LMI subgroups according to the median value of LMI in each dataset (**Figure 3A**). As expected, we found that the number of deaths increased with LMI increasing in both the training and validation cohorts (**Figure 3B**). Meanwhile, PCA was applied to demonstrate the distribution patterns of the two subgroups in two-dimensional graphs (**Figure 3C**). Finally, the KM curves for the OS of breast cancer patients showed that patients with high LMI had considerably poorer survival probability across all cohorts (**Figure 3D**), which proved the predictive accuracy of LMI in the prognoses of breast cancer.

### Assessment of the Correlation Between LMI and Clinicopathological Characteristics in BC Patients

**Figure 4:**
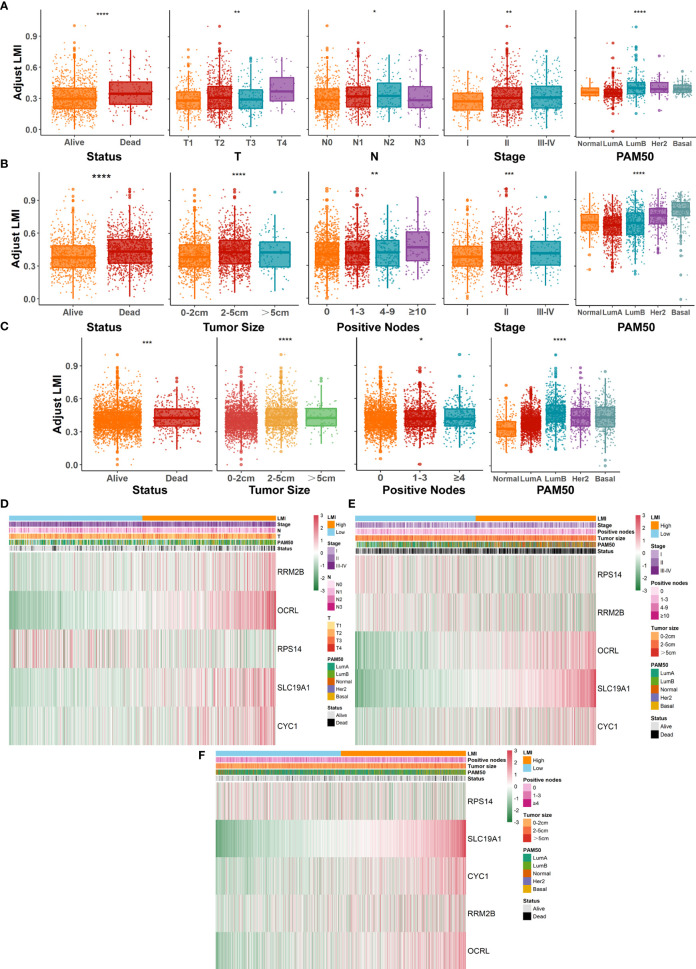
Systematic dissection of LMI and clinical parameters in BC patients. The boxplots to illustrate the correlation between LMI and different clinicopathological characteristics of BC patients in TCGA-BRCA **(A)**, METABRIC **(B)** and GSE96058 **(C)**, respectively. Correlation heatmaps of signature-included LMGs and clinicopathological features in datasets of TCGA-BRCA **(D)**, METABRIC **(E)** and GSE96058 **(F)** separately. *p < 0.05; **p < 0.01; ***p < 0.001; ****p < 0.0001.

Further evaluation of the relationship between LMI and various clinicopathological factors in breast cancer patients was also conducted. In the TCGA-BRCA training cohort (**Figure 4A**), prominent discrepancies were found between LMI and clinical characteristics, including survival status, T, N, AJCC stage, and PAM50 subtypes. Similarly, conspicuous differences were also observed in validation cohorts, containing survival status, tumor size, positive nodes, stage, and PAM50 subtypes in the METABRIC set (**Figure 4B**) and survival status, tumor size, positive nodes, and PAM50 subtypes in the GSE96058 set (**Figure 4C**). Notably, as the LMI levels were higher, the degree of clinical features tended to become more severe, which was displayed in both the training and validation sets and reconfirmed the predictive value of LMI in breast cancer. Meanwhile, heatmaps were used to demonstrate the correlation analyses between LMI-contained genes and clinicopathological features in TCGA-BRCA (**Figure 4D**), METABRIC (**Figure 4E**), and GSE96058 (**Figure 4F**) sets.

### Development and Validation of a Prognostic Nomogram Based on LMI Signature

**Figure 5:**
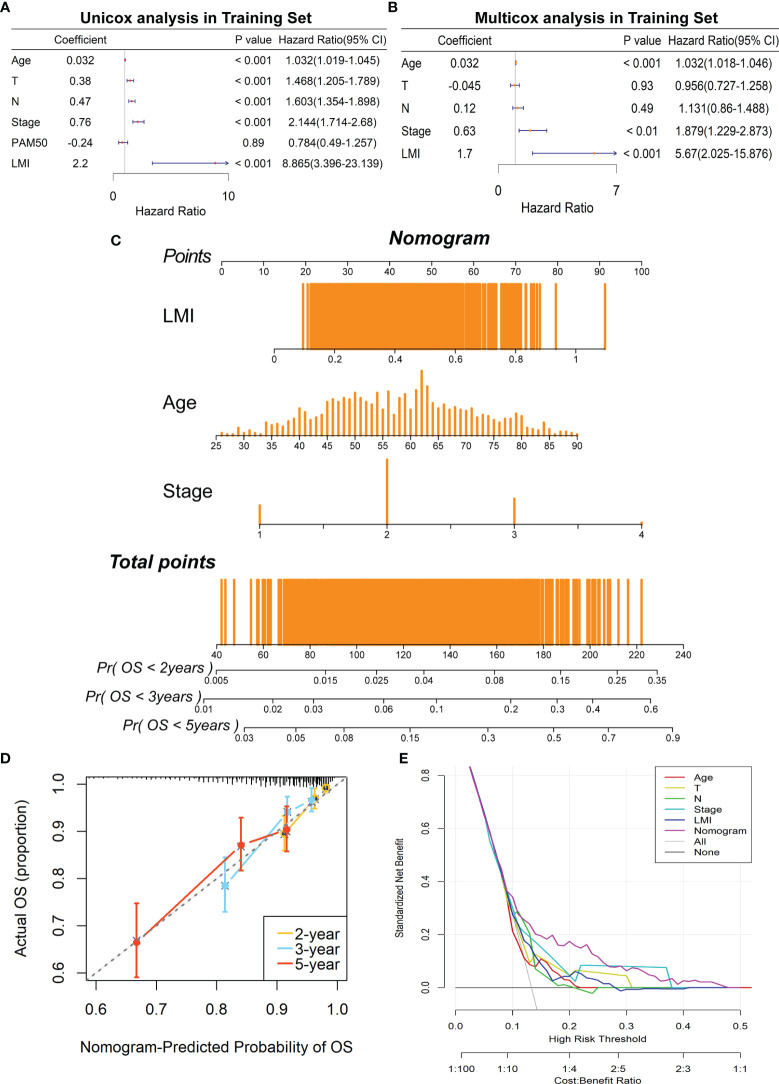
Construction of a prognostic nomogram containing LMI signature in the training set. **(A)** Univariate Cox regression analysis of age, T stage, N stage, AJCC stage, PAM50 subtypes, and LMI for OS. **(B)** Multivariate Cox regression analysis of age, T stage, N stage, AJCC stage, and LMI for OS. **(C)** Nomogram for the prediction of 2-, 3-, and 5-year survival probability. **(D)** The calibration plot to assess the consistency of predicted and actual OS based on the nomogram.**(E)** Decision curve analysis (DCA) for evaluating clinical utility of the nomogram.

Concerning whether LMI signature could be an independent prognostic predictor for breast cancer patients, univariate and multivariate Cox regression analyses were performed in the TCGA dataset. And the results illuminated that age, T stage, N stage, AJCC stage and LMI were significantly linked to OS in the univariate Cox analysis (**Figure 5A**), while only age, AJCC stage and LMI were still independent prognostic factors in the multivariate Cox analysis (**Figure 5B**). Next, a clinicopathologic nomogram, which contained LMI, age and AJCC stage, was developed to predict individual OS of 2-, 3- and 5-years (**Figure 5C**). Moreover, the calibration plot was portrayed to confirm the predictive consistency of the nomogram, which displayed great fitness (**Figure 5D**). And decision curve analysis (DCA) showed that the nomogram attained a greater clinical net benefit than any single clinical feature (**Figure 5E**). Collectively, the prognostic nomogram based on LMI signature has excellent prediction performance for OS of breast cancer patients.

### Verification of Different Biological Functions Between Two LMI Groups

**Figure 6:**
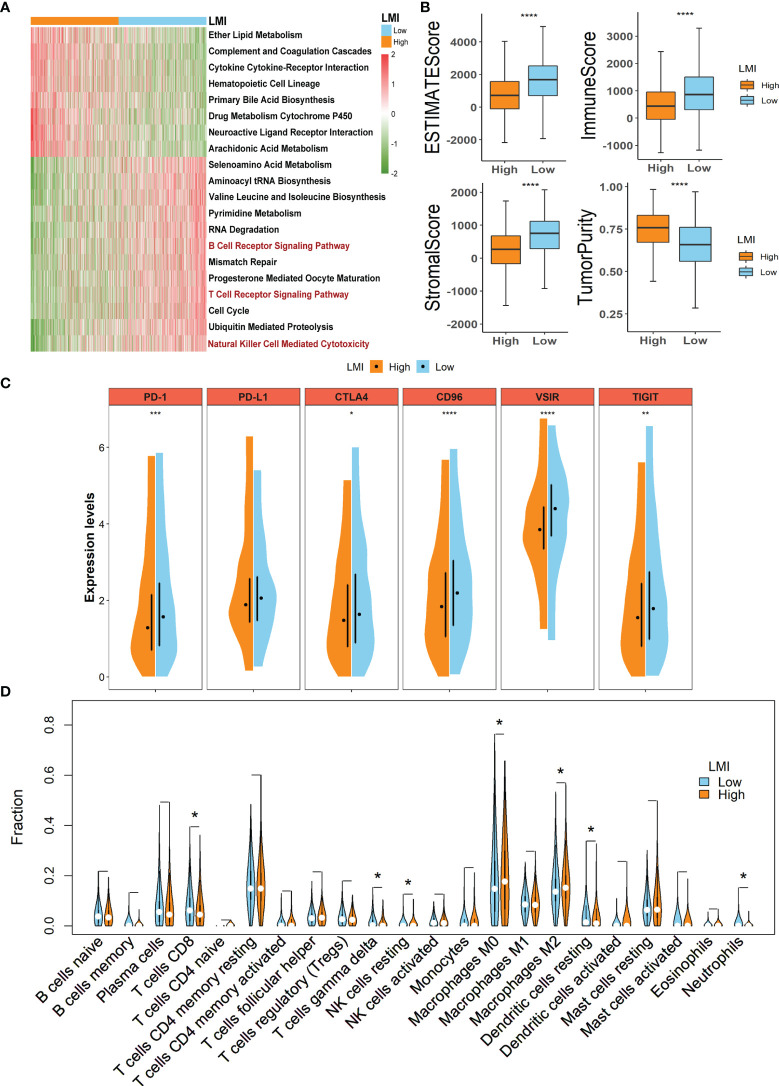
Potential implications for immunotherapy and TME landscape estimation between the high- and low-LMI groups. **(A)** Results of GSVA in TCGA-BRCA cohort to delineate the enriched pathways. **(B)** Differences of ESTIMATE score, immune score, stromal score, and tumor purity. **(C)** Expression levels of immune checkpoints. **(D)** Violin plots to display the proportions of 22 immune infiltrating cells in total BC patients. *p < 0.05; **p < 0.01; ***p < 0.001; ****p < 0.0001.

To clarify the different biological functions and signaling pathways between high- and low-LMI groups in the training set, “GSVA” enrichment analysis was exploited to detect that there are substantial differences in the metabolic pathways and immune-related pathways in the two LMI subgroups of BC (**Figure 6A**). For instance, ether lipid metabolism, cytokine cytokine-receptor interaction, and arachidonic acid metabolism were primarily enriched in the high-LMI group, while selenoamino acid metabolism, pyrimidine metabolism, B-cell receptor signaling pathway, T-cell receptor signaling pathway, and natural killer cell-mediated cytotoxicity were considerably enriched in the low-LMI group.

### Underlying Implications for Immunotherapy and TME Landscape Estimation Based on LMI

As the aforementioned result unveiled the immune-related pathways with significant differences between the high- and low-LMI groups, considerable attention was paid to the TME landscape and characteristics of the two LMI subgroups. ESTIMATE algorithm was utilized to calculate the stromal score, immune score, and ESTIMATE score to evaluate and quantify the TME, the results of which unraveled that the low-LMI group of BC obtained the higher ESTIMATE score, stromal score, and immune score together with the diminished tumor purity than the high-LMI group (**Figure 6B**). Furthermore, the comparison of expression levels of the candidate immune checkpoints within the two LMI groups manifested that PD-1, CTLA4, CD96, VSIR, TIGIT except PD-L1 were significantly augmented in the low-LMI group (**Figure 6C**), which hinted that BC patients with lower LMI might acquire a more enhanced response to immunotherapy targeting the checkpoints above. Aiming to estimate the distribution of immune infiltrating cells in the TME of BC in various LMI groups, the CIBERSORT algorithm was performed to disclose that CD8+ T cells, gamma delta T cells, resting NK cells, resting dendritic cells, and neutrophils were notably enriched in TME of the low-LMI group while macrophages M0 and M2 were markedly strengthened in the high-LMI group (**Figure 6D**). Collectively, the results above shed light on that BC patients of the lower LMI could attain an immune-activated TME, while BC patients of the higher LMI might gain an immunosuppressive TME that contributed to the immune escape of tumor cells and a worse prognosis.

**Figure 7:**
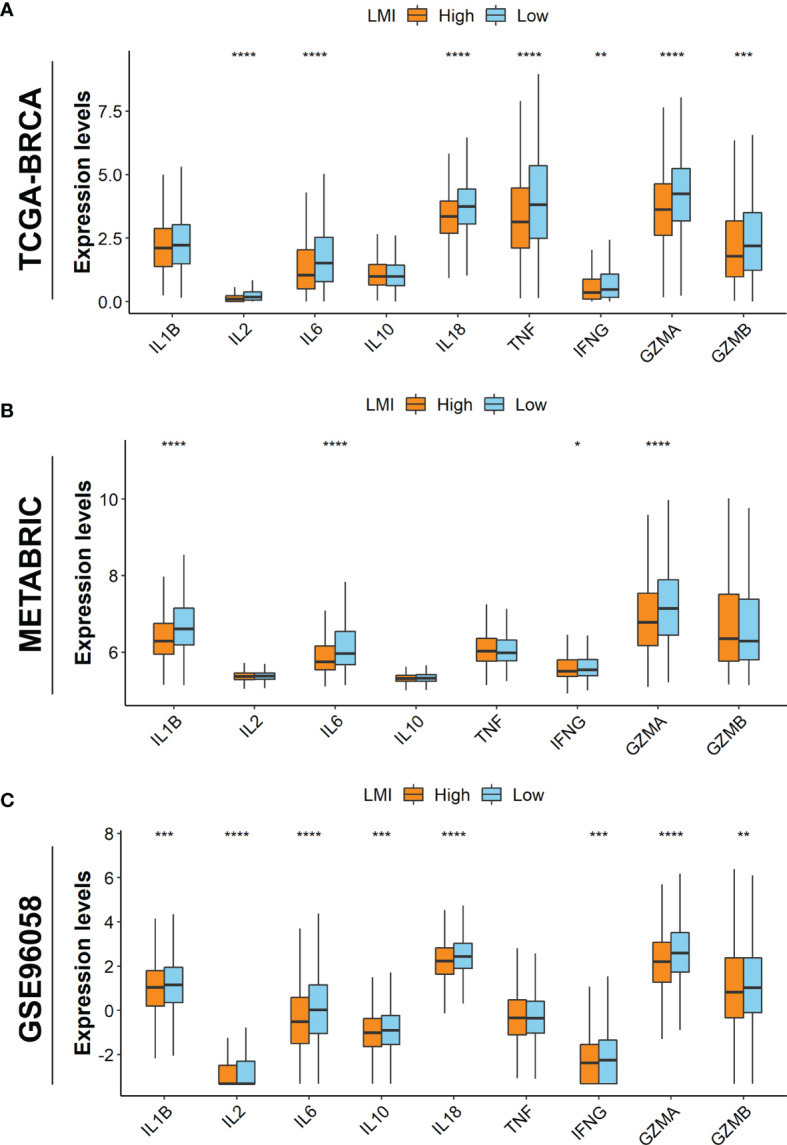
Investigations of TME cytokines. Expression levels of IL-1B, IL-2, IL-6, IL-10, IL-18, TNF, IFNG, GZMA, and GZMB in TCGA-BRCA **(A)**, METABRIC **(B)**, and GSE96058 **(C)** datasets. *p < 0.05; **p < 0.01; ***p < 0.001; ****p < 0.0001.

Additionally, it is well established that cytokines play a crucial role in the immune TME. Therefore, the boxplots in TCGA-BRCA dataset show that the expression levels of IL-2, IL-6, IL-18, TNF, IFNG, GZMA, and GZMB were promoted in the low-LMI group (**Figure 7A**). Likewise, in the METABRIC dataset, IL-1B, IL-6, IFNG, and GZMA were enhanced in the low-LMI group (**Figure 7B**). Meanwhile, IL-1B, IL-2, IL-6, IL-10, IL-18, IFNG, GZMA, and GZMB were elevated in BC patients of the low-LMI group (**Figure 7C**).

## Discussion

Despite great improvement in breast cancer treatment, some patients still have inferior outcomes after systemic therapy measures. In order to distinguish this target population, the identification of novel and valid biomarkers is needed. As considerable studies reveal that lactate has a vital role in tumorigenesis and progression, lactate metabolism has drawn increasing attention in recent years (3). For breast cancer, tumor lactate has been indicated as an unfavorable biomarker and was related to HER2 status and trastuzumab susceptibility (7, 8). However, additional data and more studies were required to support this conclusion. To provide more direct evidence for the important finding, our study integrated mRNA expression profiles of lactate metabolism-related genes and clinical variables of breast cancer patients from three independent databases to construct a predictive signature and then validate its efficacy.

In search of the most effective LMGs to establish the signature, we initially performed univariate Cox regression analyses of OS in breast cancer patients of both TCGA and METABRIC databases. Nine obtained genes were then further optimized with LASSO Cox regression analysis in TCGA-BRCA, and 5 pivotal genes were enrolled to establish the prognostic signature, namely LMI, including RPS14, SLC19A1, CYC1, RRM2B, and OCRL. The ribosomal protein S14 (RPS14), which was identified to be associated with the cancer-prone 5q-syndrome, was also found to negate c-Myc functions and promotes the proliferation and metastasis of estrogen receptor-positive breast cancer cells (28, 29). However, the result diverged from the findings shown in **Figure 2**, which may have resulted from different subtypes of breast cancer. Solute carrier family 19 member 1(SLC19A1), as the first known transporter of cGAMP and other CDNs (30), has been suggested to exert influence on cancer immunotherapy (31). Our study indicated that high expression of SLC19A1 was related to poorer survival in breast cancer, and therefore, the effects and potential functions of SLC19A1 on breast cancer initiation and progression warrant further investigation. Cytochrome c1 (CYC1), an important subunit of mitochondrial complex III, was found to be upregulated in breast cancer and might be a predictive factor assisting future patient diagnosis (32, 33), which was consistent with the results of our study. Ribonucleotide reductase M2B (RRM2B), also known as p53R2, was believed to play essential roles in DNA repair, mtDNA synthesis, and protection against oxidative stress (34) and has been reported to be associated with tumorigenesis of several cancer types, including colorectal cancer (35), hepatocellular carcinoma (36), esophageal squamous cell carcinoma (37), and breast cancer (38). As an unfavorable factor indicated in the present study, we suggested intensive research on the role of RRM2B in breast cancer. As for OCRL, the role in cancer remained to be further elucidated by experiments.

Based on the above five selected genes, a prognostic signature was established and was named LMI. To explore the prognostic value and clinical relevance of LMI, we proceeded with the following investigation. From the result of survival analyses, high LMI levels were confirmed to predict worse overall survival probability in breast cancer patients of three datasets independently. Besides, patients of each dataset were clarified into subgroups according to different clinical features, including survival status, T stage or tumor size, N stage or positive nodes, AJCC stage, PAM50 subtypes, and LMI levels of various subgroups were then compared. In general, increasing LMI value was associated with larger tumors, more metastasis lymph nodes, and a more severe AJCC stage. Nevertheless, the results of the three different cohorts were not in complete agreement with each other, and naturally, a much larger cohort should be required to confirm these findings in prospective studies. Furthermore, the LMI signature was identified as an independent prognostic indicator when adjusted for several vital clinical variables, such as age, T stage, N stage, AJCC stage, and PAM50 subtypes in the training set, through univariate and multivariate Cox regression analyses. Finally, a prognostic nomogram, which incorporated LMI value, age, and AJCC stage, was developed for predicting the OS rate of breast cancer patients in TCGA-BRCA. Calibration plots and DCA plots were also applied to assess the practicability of the prognostic nomogram, which exhibited good fitness and the potential clinical feasibility. Moreover, to investigate LMI-related molecular functions, GSVA results revealed that tumor metabolism-related signaling pathways were highly enriched in the high-LMI group, such as ether lipid metabolism, cytokine cytokine-receptor interaction, and arachidonic acid metabolism pathways, while immune-related functions were highly enriched in the low-LMI group, like B-cell receptor signaling pathway, T-cell receptor signaling pathway, and natural killer cell-mediated cytotoxicity pathways. Certainly, further studies should be warranted to elucidate clearly the complete and detailed mechanisms involved in this process.

As lactate metabolism has become a hotspot in cancer research due to its important role in TME (12), we further explored the TME landscape estimation based on LMI. Above all, ESTIMATE results presented that LMI was negatively correlated with estimate score, immune score, and stromal score but positively with tumor purity, suggesting that LMI signature could serve as a novel and potential immune indicator in breast cancer. Meanwhile, cancer immunotherapy has now become one of the pillars in the treatment of various cancer, including breast cancer (39). However, tumor-induced immune suppression, to which lactate metabolism has contributed to some extent, has been a major barrier to the effective responses of immune therapy until the present time (40). Thus, it remains especially important to find reliable biomarkers that could be used to precisely identify breast cancer patients for immunotherapy. After comparing the expression levels of common immune checkpoints between high- and low-LMI groups in breast cancer, we found that expressions of PD-1, CTLA4, CD96, VSIR, and TIGIT except PD-L1 were significantly upregulated in the low-LMI group, which hinted that breast cancer patients with lower LMI value might have had a better immunotherapy response. To some extent, the results were consistent that high lactate has contributed to immune invasion and suppressed antitumor immune responses. Subsequently, the landscape of tumor-infiltrating immune cells between high- and low-LMI groups was estimated and the result disclosed that CD8+ T cells, gamma delta T cells, resting NK cells, resting dendritic cells, and neutrophils were notably enriched in the TME of the low-LMI group while macrophages M0 and M2 were markedly strengthened in the high-LMI group. Increased lactate has been confirmed as a major contributor to acidosis in the TME, and accordingly decreased extracellular pH has been verified to weaken functions of CD8+ and CD4+ lymphocytes, including activation, cytotoxicity, chemotaxis, motility, and proliferation (41). As well as inhibiting effector T cell function, lactate concentrations in the TME favor immunosuppressive Treg development (42). As will be discussed, lactate can directly suppress the cytotoxic functions of DCs, natural killer (NK), and natural killer T (NKT) cells (12, 14). For tumor-associated macrophages (TAMs), lactate-driven TAM polarization is a vital mechanism of immune escape for cancer cells (40). Taken together, breast cancer patients with high-LMI value have unfavorable immunotherapy responses due to the deficiency of effective antitumor immune cells and the enrichment of immunosuppressive cells. In addition, cytokines, which are present in the TME, have an essential role in cancer pathogenesis and cancer therapy (43). Generally, host-derived cytokines can suppress tumor progression and tumor cells can exploit host-derived cytokines to promote development (44). Through evaluating the expression levels of several cytokines in three datasets, we concluded that IL-6, IFNG, and GZMA were highly expressed in the low-LMI group.

Strikingly, the present study provided a comprehensive analysis of LMGs in breast cancer and constructed an effective LMI signature which has implications for future studies of breast cancer immune treatment. Nevertheless, there remain some limitations that should be contemplated. All the conclusions should be further supported by experimental data.

## Conclusion

To conclude, our research established a reliable clinical signature of LMI rooted in lactate metabolism-related genes for BC patients. Additionally, the signature was unraveled as an independent prognostic factor, and a nomogram with high usability embodying LMI was generated. The latent connotations between LMI and tumor immune microenvironment were unveiled. In a nutshell, our study might support crucial preclinical significance in cancer research about lactate metabolism and immunotherapy.

## Data Availability Statement

The original contributions presented in the study are included in the article/**Supplementary Material**. Further inquiries can be directed to the corresponding authors.

## Author Contributions

All authors participated in the present study, including conception and design (ZZ and YP), data collection, data analysis, drafting or critically revising the article (LY, PT, and HS), as well as study supervision (ZZ and YP). All authors have read and approved the final version submitted.

## Funding

This study was supported by the Guangzhou Municipal Science and Technology Project (201804010132 to HS) and the National Natural Science Foundation of Guangdong Province, China (2022A1515012536 to LY).

## Conflict of Interest

The authors declare that the research was conducted in the absence of any commercial or financial relationships that could be construed as a potential conflict of interest.

## Publisher’s Note

All claims expressed in this article are solely those of the authors and do not necessarily represent those of their affiliated organizations, or those of the publisher, the editors and the reviewers. Any product that may be evaluated in this article, or claim that may be made by its manufacturer, is not guaranteed or endorsed by the publisher.

## Footnotes

1
https://portal.gdc.cancer.gov/repository

2
http://www.cbioportal.org/

3
https://www.ncbi.nlm.nih.gov/geo/

4
http://www.gsea-msigdb.org/gsea/msigdb/index.jsp

5
https://xenabrowser.net/datapages/
