# Supplementary material for: Integrative Dissection of Novel Lactate Metabolism-Related Signature in the Tumor Immune Microenvironment and Prognostic Prediction in Breast Cancer
Source: Front Oncol. 2022 Apr 27;12:874731. doi: 10.3389/fonc.2022.874731 (PMC9094627; doi:10.3389/fonc.2022.874731)

Supplementary Material

SUPPLEMENTARY FIGURE LEGEND

Supplementary Figure 1: Identification of 205 reliable lactate metabolism-related genes. (A) Venn diagram to identify 205 overlapping LMGs in TCGA-BRCA, METABRIC and GSE96058 datasets. (B) The information of 205 eligible LMGs.

A

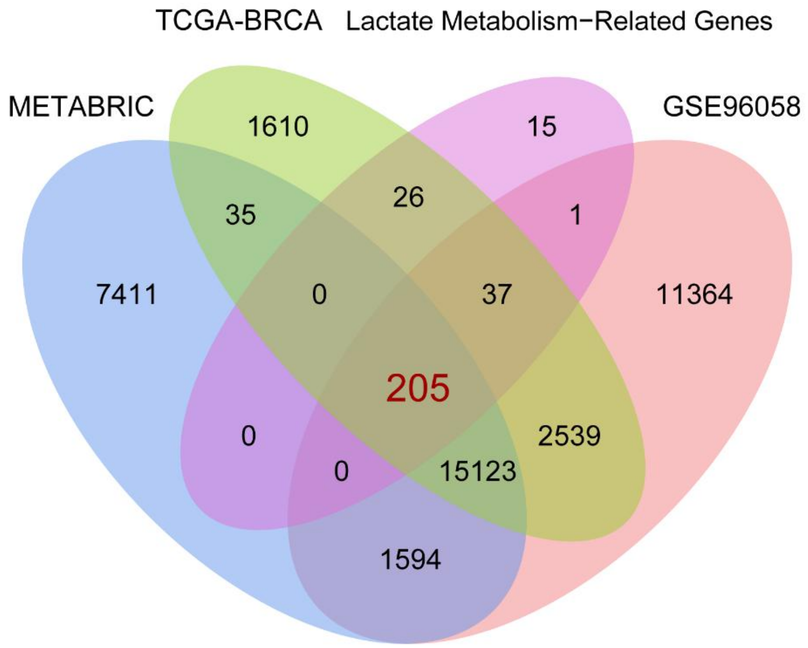

B

Supplement Table1. The information of 205 lactate metabolism-related genes								
1	COQ9	NDUFS4	PDP1	MVK	NDUFA12	WARS2	SLC13A3	LYRM7
2	FBXL4	SDHA	AIFM1	LDHA	PYGL	SIL1	PC	FKRP
3	RARS2	SPP1	GYS2	PITRM1	SCO1	RNASEH1	CLPB	ACAT1
4	KY	AARS2	SLC5A12	NDUFAF2	TCIRG1	HTRA2	SLC16A8	B3GALNT2
5	TIMM22	LIPT1	MYC	NDUFV2	CHEK2	PHKG2	SLC16A7	ALDH4A1
6	MRPL3	CYP27A1	CA5A	BCS1L	INPP5K	CD46	PNPLA8	DLD
7	PNPT1	COX8A	POMT1	NDUFB8	JAK2	TK2	MECP2	YARS2
8	NDUFS2	SLC25A13	PLEC	LDHC	TMEM126B	NDUFA9	COX4I1	PLA2G6
9	MRPS28	RHCE	SLC25A42	LIAS	TMEM70	NDUFV1	NFS1	SLC25A10
10	SLC16A1	MRPS34	NDUFS8	MRPL44	NDUFA10	SOD1	PDHX	NARS2
11	UQCRC2	SDHB	NDUFA4	HMGCL	PNPO	OGDH	NDUFA8	RB1
12	MPV17	COL4A1	DNM1L	LDHD	MRPS14	CFH	GFM1	LIPA
13	RPS14	PNPLA2	POLG	GATA1	C1QBP	NDUFAF1	TUFM	ISCA1
14	FARS2	NDUFB3	COX16	EMB	SLC25A4	LDHAL6B	MTO1	HBB
15	SFXN4	MRPL12	CARS2	RHAG	COX6B1	PIGA	SURF1	NGLY1
16	SLC25A19	ADAMTS1	SLC19A1	DAG1	MIPEP	CHCHD10	CYC1	VPS13A
17	TET2	SLC16A3	COX6A2	ECHS1	NDUFA13	UQCRQ	TSFM	NDUFS3
18	RMND1	POLG2	RHD	EARS2	LRPPRC	CPT2	RRM2B	LDHB
19	FASTKD2	MDH2	NDUFB11	SLC39A8	TP53	TXN2	NDUFAF3	MTFMT
20	LYST	ACAD9	TACO1	PDSS2	IRAK1	LONP1	GOT2	TARS2
21	SLC7A7	COX5A	GTPBP3	SUCLG1	GFM2	SERAC1	HLA-DRB1	LIPT2
22	HSD17B10	NDUFAF4	OCRL	PDSS1	NDUFA1	PMPCB	TRMT5	HS6ST2
23	NDUFC2	CFI	SLC25A3	DGUOK	COX10	ISCU	FOXRED1	FLI1
24	COG8	ACADM	STAT4	PUS1	UQCRB	POMGNT1	ATAD3A	NDUFB10
25	SYNJ1	ATPAF2	PDHA1	GAA	SLC25A26	NDUFA11	CALR	MRPS22
26	ACAT2	POMT2	KCNN4	NDUFS7	COQ2			

Supplementary Figure 2. Expression level of SLC19A1 and RPS14 in human breast cancer cell lines. (A) Expression level of SLC19A1. (B) Expression level of RPS14.

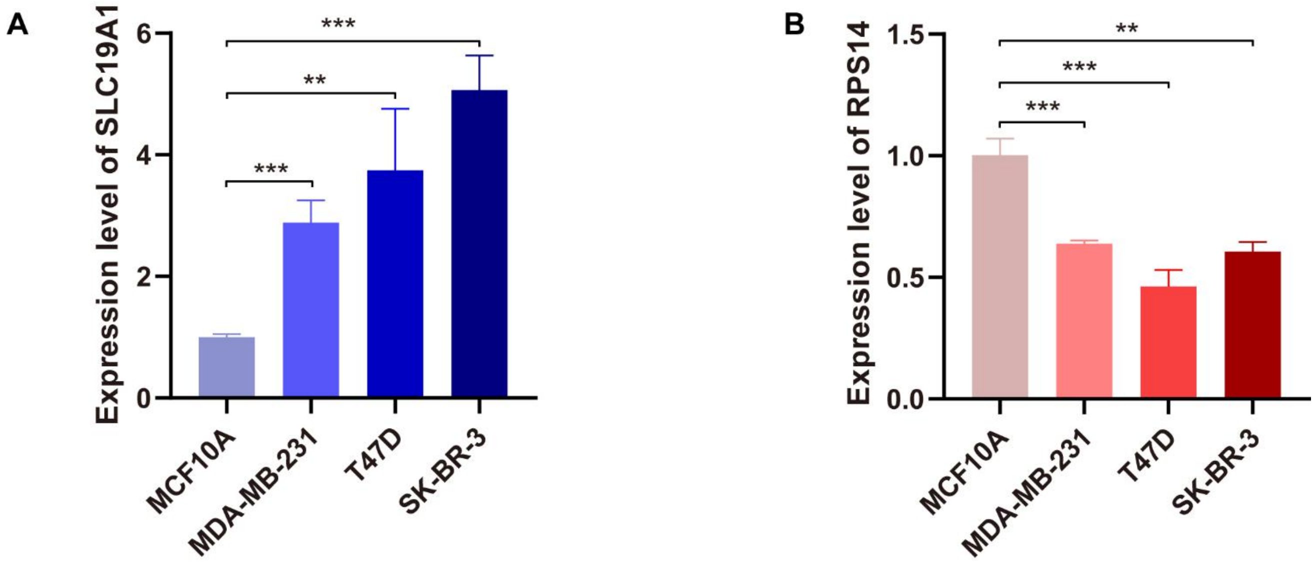

Supplementary Figure 3. ROC analysis of LMI signature in different subtypes in TCGA-BRCA. (A) Result of Basal subtype. (B) Result of non-Basal subtype.

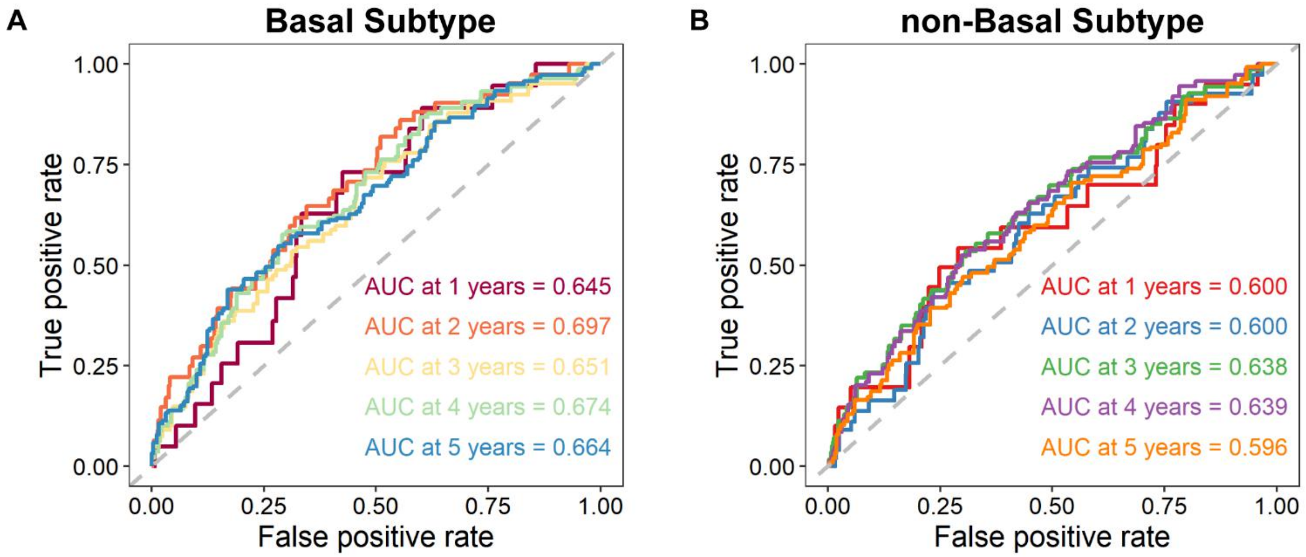

Supplement: Supplementary file 1 [file DataSheet_1.pdf]
