# Supplementary material for: Integrative Dissection of Novel Lactate Metabolism-Related Signature in the Tumor Immune Microenvironment and Prognostic Prediction in Breast Cancer
Source: Front Oncol. 2022 Apr 27;12:874731. doi: 10.3389/fonc.2022.874731 (PMC9094627; doi:10.3389/fonc.2022.874731)
Supplement: Supplementary file 4 [file Table_3.docx]

Supplementary Material

**SUPPLEMENTARY TABLE**

**Supplementary Table S3**. Primers sequences were shown.

| **Supplementary Table S1. Primers for qRT-PCR detection** | | |
| --- | --- | --- |
| SLC19A1 | Forward | CTCAGCTTCGTGTCGGTGT |
|  | Reverse | AGCGAGATGTAGTTGAGCGTG |
| RPS14 | Forward | CCATGTCACTGATCTTTCTGGC |
|  | Reverse | TCATCTCGGTCTGCCTTTACC |
| β-actin | Forward | CATGTACGTTGCTATCCAGGC |
|  | Reverse | CTCCTTAATGTCACGCACGAT |
